# Comparison of Open versus Laparoscopic Approaches in Salvage Hepatectomy for Recurrent Hepatocellular Carcinoma after Radiofrequency Ablation

**DOI:** 10.3390/medicina59071243

**Published:** 2023-07-04

**Authors:** Yeshong Park, Jai Young Cho, Ho-Seong Han, Yoo-Seok Yoon, Hae Won Lee, Boram Lee, MeeYoung Kang, Jinju Kim

**Affiliations:** Department of Surgery, Seoul National University Bundang Hospital, Seoul National University College of Medicine, Seoul 03080, Republic of Korea82637@snubh.org (M.K.);

**Keywords:** hepatocellular carcinoma, radiofrequency ablation, salvage hepatectomy, laparoscopic liver resection

## Abstract

*Background and Objectives*: Although radiofrequency ablation (RFA) is widely used as an effective local treatment for hepatocellular carcinoma (HCC), evidence on salvage hepatectomy for local recurrence after RFA is limited. This study aims to compare open and laparoscopic approaches in salvage hepatectomy for recurrent HCC after RFA. *Materials and Methods*: Among patients who underwent hepatectomy between January 2004 and August 2022 at a single tertiary referral center, 55 patients who underwent salvage hepatectomy for marginal recurrence after RFA were selected. An open approach was used in 23 (41.8%) patients, while 32 (58.2%) patients underwent laparoscopic surgery. Short-term and long-term outcomes were compared between the two groups. *Results*: Major hepatectomy was more often performed in the open group (9 [39.1%] vs. 4 [12.5%], *p* = 0.022). Intraoperative blood loss was also greater in the open group (450 (325–750) vs. 300 (200–600), *p* = 0.034). Operation time (*p* = 0.144) and postoperative morbidity rates (*p* = 0.639) were similar, and there was no postoperative mortality in either group. Postoperative hospital stay was significantly longer in the open group compared to the laparoscopy group (8 (6–11) days vs. 5 (4–7) days, *p* = 0.028). The 1-, 3-, and 5-year disease-free survival rates showed no difference between the two groups (44.6% vs. 62.5%, 16.5% vs. 13.5%, and 8.3% vs. 13.5%, respectively; *p* = 0.154). The 1-, 3-, and 5-year overall survival rates between the two groups were also similar (85.7% vs. 96.8%, 79.6% vs. 86.0%, and 79.6% vs. 79.4%, respectively; *p* = 0.480). *Conclusions*: Laparoscopic salvage hepatectomy shows oncologic outcomes comparable to the open approach with faster postoperative recovery rates. Considering that recurrence rates are high after RFA, the laparoscopic approach should be considered as a first-line option in selected patients.

## 1. Introduction

Treatment selection for hepatocellular carcinoma (HCC) is based on various factors including disease stage, underlying liver condition, and the performance status of the patient. Local ablation techniques including radiofrequency ablation (RFA) are accepted as curative therapeutic options for very early and early-stage HCC [1,2,3]. However, marginal recurrence after RFA has been reported in 2% to 36% of patients, and high recurrence rates are generally known to affect long-term survival after RFA [4,5].

Most patients experiencing recurrence after RFA are treated with repeated local ablation or transcatheter arterial chemoembolization (TACE) [6]. Surgical treatment for recurred tumors, referred to as salvage hepatectomy, has also been reported as an acceptable treatment option [4,7,8,9]. Previous studies found the survival benefit of salvage hepatectomy to be comparable to primary hepatic resection with similar overall survival rates [4,7]. Yet the technical feasibility of salvage hepatectomy has been challenged; RFA procedures might cause dense adhesions that render the approach for liver mobilization extremely difficult, and more extensive resections might be necessary due to advanced-stage tumors [7,8].

Since its introduction, laparoscopic liver resection has been associated with better short-term outcomes and similar oncologic survival compared to the open approach [10,11,12]. Due to advances in minimally invasive techniques and surgical strategies, the indication for laparoscopic surgery has been extended to tumors in difficult locations, underlying advanced cirrhosis, and elderly patients with multiple comorbidities [13,14,15]. Yet the role of laparoscopic surgery in salvage hepatectomy for local recurrence after RFA has never been addressed. The aim of this study was to compare the short-term and long-term outcomes of open and laparoscopic salvage hepatectomy for recurrent HCC after RFA.

## 2. Materials and Methods

### 2.1. Study Population

**Figure 1:**
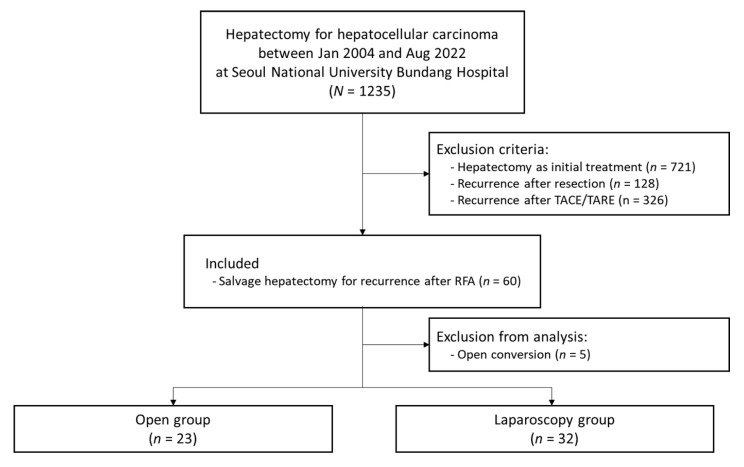
Flowchart diagram for patient selection.

Between January 2004 and August 2022, 1235 patients underwent hepatectomy for HCC at Seoul National University Bundang Hospital (SNUBH), a tertiary referral hospital in Korea. Among them, 60 consecutive patients who underwent salvage hepatectomy for marginal recurrence after RFA were selected. Five patients who underwent open conversion were excluded from the analysis. Finally, 23 patients who underwent open salvage hepatectomy and 32 patients who received laparoscopic salvage hepatectomy were compared (Figure 1). This study was approved by the institutional review boards of SNUBH and conducted in compliance with the STROBE guidelines for cohort studies.

### 2.2. Data Collection and Definitions

Demographic information, information related to operative data, information on pathological features, and survival data were collected from medical records. The terminology used for the hepatectomy procedures was based on Couinaud’s classification [16]. Major hepatectomy was defined as the resection of three or more liver segments, and minor hepatectomy was defined as sectionectomy, segmentectomy, or non-anatomical liver wedge resection. Estimated blood loss was based on visual estimation at the end of surgery by both the surgeon and the anesthesiologist. Morbidities were graded by the Clavien–Dindo classification system [17]. In-hospital death was defined as death at any time during hospital stay.

### 2.3. Surgical Procedure

The decision on surgical approach was made individually based on tumor characteristics including size, number, and location and patient factors including liver cirrhosis, underlying disease, and history of previous abdominal operations. Open and laparoscopic salvage hepatectomies were performed by the same team with standardized operation procedures that were not influenced by the surgical approach. In open hepatectomy, the patient was placed in the supine position under general anesthesia. An upper midline incision with a right extension was used. Intraoperative ultrasound examinations were utilized to confirm the location and size of the tumor and its location in relation to major vessels. A Cavitron ultrasonic surgical aspirator (CUSA; Valleylab, Boulder, CO, USA) was used for liver parenchymal transection. Hemostasis was pursued through the use of monopolar or bipolar electrocautery, argon beam, or sutures. Abdominal drains were routinely placed at the hepatic resection margin.

In laparoscopic hepatectomy, the patient was placed in the lithotomy position. A subumbilical 12 mm trocar was used as the camera port. All operations were performed with a three-dimensional flexible tip laparoscope. Three to four additional working ports were placed in consideration of the tumor’s location and size. Ultrasound examination was performed in the same manner as for the open approach. An ultrasonic dissector (Harmonic; Ethicon, Cincinnati, OH, USA) was used for adhesiolysis, liver mobilization, and superficial liver parenchymal transection; for deeper dissection, CUSA was used as in open hepatectomy. Hemostasis was performed with monopolar electrocautery, metal clips, or sutures. The liver specimen was delivered through an extension of the subumbilical incision. Abdominal drains were placed using the 5 mm trocar sites at the hepatic resection margin.

### 2.4. Follow-Up

All of the patients underwent regular follow-up with clinical examination, measurement of serum tumor markers including α-fetoprotein (AFP) and des-γ-carboxyprothrombin (DCP), and imaging studies including computed tomography (CT) or gadoxetic-acid-enhanced magnetic resonance imaging (MRI). Recurrence was defined as the appearance of a new lesion with radiologic features typical of HCC. The median time to recurrence after RFA was 9 months. Disease-free survival was defined as the interval between the operation and the date of first recurrence. Overall survival was defined as the interval between the operation and the date of cancer-related death or the last follow-up. The median follow-up duration was 33 months.

### 2.5. Statistical Analysis

All statistical analyses were performed using SPSS (version 25.0, IBM Inc., Armonk, NY, USA). Categorical data were expressed as frequency (percentage). Continuous variables were expressed as the mean ± standard deviation for normally distributed variables or as the median (interquartile range) for non-normally distributed variables. Continuous variables were compared using Student’s *t*-test or the Mann–Whitney U test; categorical variables were compared using Pearson’s chi-square test or Fisher’s exact test. Survival analysis was conducted with the Kaplan–Meier method and log-rank test. Prognosis factors for survival were investigated using univariable and multivariable Cox regression analyses. All *p*-values were two-sided, and *p* < 0.05 was considered statistically significant.

## 3. Results

### 3.1. Patient Characteristics

The clinical characteristics of the study participants are summarized in Table 1. The open and laparoscopic salvage hepatectomy groups showed no statistically significant difference in age, sex, body mass index (BMI), and operation history. Baseline liver functions including Child–Pugh class, Model for End-Stage Liver Disease (MELD) score, platelet count, prothrombin time, and serum albumin level were similar between the two groups. Tumor marker levels also showed no difference.

### 3.2. Operative Parameters

The operative parameters were compared between the open and laparoscopy groups (Table 2). Major hepatectomy was more frequently performed during open salvage hepatectomy (9 [39.1%] vs. 4 [12.5%], *p* = 0.049). There was no difference in anatomical resection rate, operation time, and duration of the Pringle maneuver. The open conversion rate in the laparoscopic group was 13.5% (5/37), with severe adhesion in three patients, intraoperative vital instability in one patient, and difficulty in securing the resection margin in one patient. Estimated blood loss was greater in the open group (450 (325–750) vs. 300 (200–600), *p* = 0.034), but the intraoperative transfusion rates were similar between the two groups.

### 3.3. Pathologic Features and Postoperative Outcomes

When pathological data of the two groups were compared, there was no difference in tumor location, tumor number, tumor grade, vascular invasion rate, or margin status (Table 3). The tumor size was larger in the open salvage hepatectomy group (3.0 [1.9–3.5] vs. 2.0 [1.2–3.0], *p* = 0.049). In the analysis of postoperative outcomes, no statistically significant difference in postoperative complication rates or intensive care unit admission was found. There were no in-hospital deaths in either group. Postoperative hospital stay was significantly shorter in the laparoscopy group (8 [6,7,8,9,10,11] vs. 5 [4,5,6,7], *p* = 0.028).

### 3.4. Survival Outcomes

**Figure 2:**
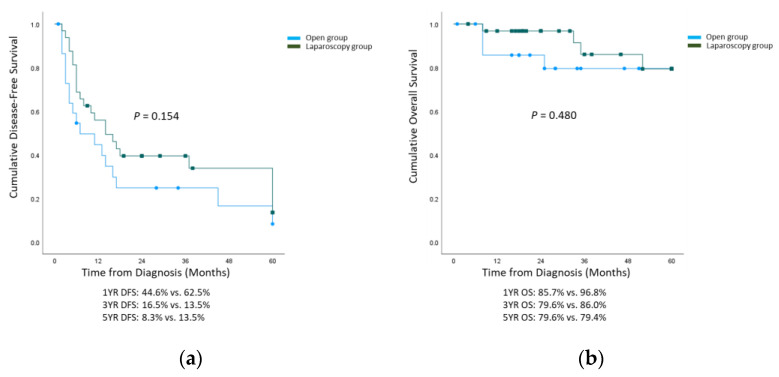
Kaplan–Meier survival curves for disease-free and overall survival. (**a**) 5-year disease-free survival and (**b**) 5-year overall survival.

The overall 1-, 3-, and 5-year DFS rates of the study population were 48.7, 32.5%, and 26.2%, respectively. The recurrence rates showed no statistically significant difference between the open and laparoscopic salvage hepatectomy groups for either local recurrence (11 [47.8%] vs. 21 [65.6%], *p* = 0.187) or systemic recurrence (11 [47.8%] vs. 9 [28.1%], *p* = 0.134). There was no difference in the 1-, 3-, and 5-year DFS rates between the two groups (44.6% vs. 62.5%, 16.5% vs. 13.5%, and 8.3% vs. 13.5%, respectively; *p* = 0.154). The overall 1-, 3-, and 5-year OS rates of the study population were 93.0%, 81.9%, and 78.0%, respectively. The cancer-related death rates were similar between the two groups (5 [21.7%] vs. 8 [25.0%], *p* = 0.779). The difference in the 1-, 3-, and 5-year overall survival rates between the two groups was also not statistically significant (85.7% vs. 96.8%, 79.6% vs. 86.0%, and 79.6% vs. 79.4%, respectively; *p* = 0.480). The cumulative disease-free and overall survival curves are shown in Figure 2.

### 3.5. Risk Factor Analysis for Recurrence after RFA

Cox regression analysis was conducted to analyze risk factors for recurrence after RFA (Table 4). Tumor number was the only significant predictor for recurrence (hazard ratio [HR] 3.05, *p* = 0.009). In the univariable Cox regression analysis of risk factors for cancer-related death, Child–Pugh class, tumor number, tumor grade, and vascular invasion were found to be significant. In the multivariable analysis, tumor number (HR 8.34, *p* = 0.009), tumor grade (HR 17.98, *p* = 0.008), and vascular invasion (HR 8.19, *p* = 0.014) remained as prognostic factors.

## 4. Discussion

HCC is the fourth leading cause of cancer mortality worldwide and the second most common cause of cancer mortality in Korea [18]. RFA is an effective treatment strategy for early HCC, especially in patients with limited liver functional reserve [19]. However, post-RFA recurrence is relatively common, and locally recurrent tumors show more aggressive behavior compared to primary tumors [20,21,22]. Studies on the treatment strategies and long-term outcomes of local recurrence after RFA are limited [6]. Previous studies have shown that salvage hepatectomy could be a therapeutic option for recurrence after RFA, yet most results are based on retrospective cohort studies from single centers with limited sample sizes [4,7,8,9]. Due to such limitations, the effect of the surgical approach in salvage hepatectomy has never been addressed. To our knowledge, this is the first study to compare short-term and long-term outcomes of open and laparoscopic salvage hepatectomy for local recurrence after RFA.

Salvage hepatectomy for local recurrence after RFA has been associated with more technical difficulties compared to primary liver resection. One of the advantages of RFA over surgical resection is its less invasive nature, which allows for repeated procedures in case of recurrence; for such reasons, patients referred for salvage hepatectomy are likely to be those for whom repeated RFA was technically difficult [23,24]. Also, RFA might cause dense adhesions around the liver, which renders liver mobilization and approach to the tumor even more challenging. Previous studies showed that salvage hepatectomy was associated with longer operation times, more intraoperative blood loss, and higher transfusion rates compared to patients undergoing primary liver resection [4,7]. Yamashita et al. also found postoperative morbidity rates to be higher in the salvage hepatectomy group compared to a matched control group who underwent hepatectomy as initial treatment [8]. However, when salvage hepatectomy for recurrence after RFA was compared with secondary liver resection for recurrence after surgery, short-term outcomes including operative data and postoperative complications showed no statistically significant difference [25].

In all previous studies, salvage hepatectomy for recurrence after RFA was performed exclusively by the open approach. Therefore, the effect of the surgical approach on postoperative outcomes after salvage hepatectomy could not be analyzed. In the current study, 58.2% of all patients received laparoscopic surgery. Short-term outcomes including intraoperative blood loss, transfusion rates, operation time, and postoperative complication rates were comparable to previous studies. We also found that the laparoscopic approach was associated with less blood loss and shorter postoperative hospital stays. These results suggest that laparoscopic salvage hepatectomy is safe and technically feasible if performed by experienced surgeons. Yet the tumor size was larger in the open group, and major hepatectomy was more often performed by the open approach. Decision on the surgical approach, either open or laparoscopic, was made independently by the individual surgeon based on various factors including tumor size and location. It is possible that operators tended to use the open approach more often when major liver resection was necessary. This difference in tumor size and major hepatectomy rate could have influenced the postoperative outcomes including operation time, intraoperative blood loss, and postoperative hospital stay. Still, the results of the current study suggest that laparoscopic salvage hepatectomy could be performed safely in patients who are determined to be suitable for minimally invasive liver resection. In patients with large recurrent tumors requiring major hepatectomy or concurrent extrahepatic resection, open surgery should be considered. Further large-scale prospective studies are necessary to establish practical guidelines for performing laparoscopic salvage hepatectomy.

Reports on the long-term survival outcomes after salvage hepatectomy for recurrence after RFA vary between studies. Sugo et al. found no significant difference in overall survival between patients who underwent salvage hepatectomy and those who underwent primary hepatectomy, with a 5-year survival rate of 67% in the salvage hepatectomy group [4]. Yet the 1-, 3-, and 5-year disease-free survival rates were significantly worse in the salvage group. Another study from Japan found the 5-year overall survival rate of patients undergoing salvage hepatectomy to be 58.3%, which was equivalent to the Japanese nationwide survey of HCC [9]. On the contrary, Yamamoto et al. reported that cumulative survival rates were only 9.5% at 5 years in patients undergoing salvage hepatectomy, which was worse than the known survival rates after surgery as the initial treatment in Japanese HCC patients [26]. An Italian study on salvage hepatic resection for incomplete ablation of primary and secondary liver tumors reported that both 2-year disease-free survival rates (salvage hepatectomy 28.5% vs. primary hepatectomy 83.1%, *p* = 0.003) and overall survival rates (salvage hepatectomy 44.4% vs. primary hepatectomy 87.1%, *p* < 0.001) were worse in the salvage hepatectomy group [7].

In the current study, the 5-year overall survival rate of patients undergoing salvage hepatectomy was 78.0%, which was superior to previous studies. There was no difference in overall survival between the open and laparoscopy groups, which suggested that the survival outcomes were comparable between the two approaches. However, in accordance with previous literature, recurrence after salvage hepatectomy was relatively common. Systemic recurrence rates were especially high, which reflected the aggressive behavior of recurrent HCC after RFA. Disease-free survival rates between open and laparoscopic salvage hepatectomy showed no statistically significant difference. This result suggested that the relatively high recurrence rates after salvage hepatectomy should be attributed to the tumor biology itself, rather than the surgical approach.

Local recurrence after RFA has shown a correlation with various clinicopathological factors. Previous studies found that large tumor size, multiple nodules, high serum tumor marker levels, insufficient tumor margin, tumor location near the liver surface, and decreased expertise of the performing physician were associated with an increased risk of local recurrence [20,21,27,28,29,30,31]. In the current study, we found tumor number to be the only independent risk factor. The surgical approach had no prognostic effect, which suggested that laparoscopic salvage hepatectomy can be performed with favorable oncologic outcomes. Considering the relatively high recurrence rates after salvage hepatectomy, there is a high possibility that these patients might need additional treatment including local ablation, TACE, or even repeated surgical resection. In such cases, patients are likely to benefit from laparoscopic surgery, which allows for faster recovery, lower morbidity rates, and a higher chance for safe reoperation.

This study has certain limitations. First, this was a retrospective study conducted at a single institution. Second, the decision on the surgical approach was made by the individual surgeon, considering both patient factors and tumor characteristics. Although the baseline characteristics between the open and laparoscopy groups showed no statistically significant difference, it is possible that a selection bias existed between the two groups. Future large-scale prospective studies are necessary to further validate the effect of the surgical approach in salvage hepatectomy for local recurrence after RFA.

## 5. Conclusions

Salvage hepatectomy is an acceptable treatment option for recurrence after RFA with an overall survival benefit. Laparoscopic salvage hepatectomy shows oncologic outcomes comparable to the open approach with less intraoperative blood loss and faster postoperative recovery. Considering that recurrence rates are high after RFA and repetitive treatment may be necessary, the laparoscopic approach should be considered as a safe and feasible option in patients eligible for surgical resection.

## Figures and Tables

**Table 1:** Baseline characteristics of the study participants.

	Open (*n* = 23)	Laparoscopy (*n* = 32)	Total (*n* = 55)	*p*-Value
Age (years)	61 (53–69)	63 (54–68)	62 (54–68)	0.755
Sex (male:female)	21:2	28:4	49:6	0.999
BMI (kg/m^2^, mean ± SD)	24.0 ± 3.6	24.9 ± 2.6	24.6 ± 3.1	0.276
Hypertension	11 (47.8)	13 (40.6)	24 (43.6)	0.798
Diabetes	5 (21.7)	12 (37.5)	17 (30.9)	0.341
Cardiovascular disease	1 (4.3)	1 (3.1)	2 (3.6)	>0.999
Hepatitis B	18 (78.3)	27 (84.4)	45 (81.8)	0.726
Hepatitis C	4 (17.4)	2 (6.3)	6 (10.9)	0.223
Alcoholic	3 (13.0)	5 (15.6)	8 (14.5)	>0.999
Previous abdominal operation	8 (34.8)	12 (37.5)	20 (36.4)	>0.999
Child–Pugh class				0.418
A	22 (95.7)	32 (100)	54 (98.2)	
B	1 (4.3)	0	1 (1.8)	
MELD score	7.2 (6.8–8.4)	7.2 (6.8–8.2)	7.2 (6.8–8.4)	0.746
Platelet count (10^4^/uL)	180 (100–242)	158 (117–194)	161 (113–226)	0.379
Prothrombin time (INR)	1.03 (1.01–1.11)	1.05 (1.00–1.10)	1.04 (1.01–1.10)	0.850
Total bilirubin (mg/dL)	0.76 (0.50–1.11)	0.71 (0.62–1.10)	0.71 (0.60–1.10)	0.374
Serum albumin (g/dL)	4.3 (4.1–4.7)	4.3 (3.9–4.5)	4.3 (4.0–4.6)	0.276
AFP (ng/mL)	18.5 (2.8–137.5)	4.5 (3.2–51.0)	7.1 (3.0–59.3)	0.256
DCP (AU/mL)	27 (19–65)	24 (16–77)	25 (17–69)	0.836

BMI, body mass index; SD, standard deviation; MELD, Model for End-Stage Liver Disease; INR, international normalized ratio; AFP, alpha-fetoprotein; DCP, des-gamma-carboxy prothrombin. The values are presented as the median (interquartile range) or *n* (%) unless otherwise indicated.

**Table 2:** Comparison of operative parameters.

	Open (*n* = 23)	Laparoscopy (*n* = 32)	Total (*n* = 55)	*p*-Value
Operative extent				0.049
Major resection	9 (39.1)	4 (12.5)	13 (23.6)	
Minor resection	14 (60.9)	28 (87.5)	42 (76.4)	
Anatomical resection	12 (52.2)	11 (34.4)	23 (41.8)	0.297
Deviation from the initial plan				0.604
More extensive resection	10 (43.5)	10 (31.3)	20 (36.4)	
Less extensive resection	1 (4.3)	1 (3.1)	2 (3.6)	
Operation time (min)	230 (163–308)	153 (108–293)	220 (125–305)	0.144
Pringle time (min)	20 (15–30)	40 (23–60)	30 (15–45)	0.111
Estimated blood loss (cc)	450 (325–750)	300 (200–600)	350 (300–700)	0.034
RBC transfusion	3 (13.0)	3 (9.4)	6 (10.9)	>0.999

RBC, red blood cell. The values are presented as median (interquartile range) or *n* (%) unless otherwise indicated.

**Table 3:** Comparison of pathological features and postoperative outcomes.

	Open (*n* = 23)	Laparoscopy (*n* = 32)	Total (*n* = 55)	*p*-Value
Tumor location				0.655
Anterolateral	16 (69.6)	24 (75.0)	40 (72.7)	
Posterosuperior	7 (30.4)	8 (25.0)	15 (27.3)	
Tumor number	1 (1–1)	1 (1–1)	1 (1–1)	0.592
Tumor size (cm)	3.0 (1.9–3.5)	2.0 (1.2–3.0)	2.6 (1.5–3.2)	0.049
Edmonson grade				0.555
1	0	1 (3.4)	1 (1.8)	
2	8 (40.0)	8 (27.6)	16 (29.1)	
3	8 (40.0)	16 (55.2)	24 (43.6)	
4	4 (20.0)	4 (13.8)	8 (14.5)	
Vascular invasion				
Macrovascular	5 (21.7)	2 (6.9)	7 (12.7)	0.251
Microvascular	11 (47.8)	10 (34.5)	21 (38.2)	0.491
Margin status				>0.999
R0	20 (87.0)	26 (89.7)	46 (88.5)	
R1	3 (13.0)	3 (10.3)	6 (11.5)	
Liver cirrhosis	10 (43.5)	17 (53.1)	27 (49.1)	0.480
Complication	4 (17.4)	3 (9.4)	7 (13.2)	0.639
Angina	0	1 (3.1)	1 (1.7)	
Pleural effusion	2 (8.7)	0	3 (5.0)	
Pulmonary thromboembolism	1 (4.3)	0	1 (1.7)	
Bile leakage	2 (8.7)	2 (6.2)	5 (8.3)	
Post-hepatectomy liver failure	1 (4.3)	0	1 (1.7)	
Ileus	0	1 (3.1)	1 (1.7)	
Clavien–Dindo grade ≥ IIIa complication	3 (14.3)	1 (3.1)	4 (7.5)	0.289
Intensive care unit stay	1 (4.8)	3 (9.4)	4 (7.5)	0.999
In-hospital death	0	0	0	-
Postoperative hospital stay (day)	8 (6–11)	5 (4–7)	6 (5–9)	0.028
Follow-up (months)	28 (16–95)	36 (19–74)	33 (18–74)	0.999
Recurrence				
Local recurrence	11 (47.8)	21 (65.6)	32 (58.2)	0.187
Systemic recurrence	11 (47.8)	9 (28.1)	20 (36.4)	0.134
Cancer-related death	5 (21.7)	8 (25.0)	13 (23.6)	0.779

The values are presented as the median (interquartile range) or *n* (%) unless otherwise indicated.

**Table 4:** Cox regression analyses for recurrence and cancer-related death after RFA.

	Disease-Free Survival		Overall Survival			
	Univariable		Univariable		Multivariable	
	HR (95% CI)	*p*-Value	HR (95% CI)	*p*-Value	HR (95% CI)	*p*-Value
Sex						
Male	Ref.		Ref.			
Female	0.86 (0.30–2.42)	0.768	0.72 (0.09–5.53)	0.751		
Age (years)						
<60	Ref.		Ref.			
≥60	1.41 (0.71–2.80)	0.332	1.29 (0.42–3.96)	0.655		
Child–Pugh class						
A	Ref.		Ref.		Ref.	
B	4.44 (0.57–34.37)	0.154	11.58 (1.29–104.25)	0.029	7.59 (0.62–92.41)	0.112
AFP (ng/mL)						
<200	Ref.		Ref.			
≥200	0.94 (0.33–2.67)	0.912	1.45 (0.32–6.53)	0.633		
Operative method						
Open	Ref.		Ref.			
Laparoscopic	0.62 (0.32–1.18)	0.145	0.94 (0.31–2.88)	0.913		
Operative extent						
Minor resection	Ref.		Ref.			
Major resection	0.84 (0.38–1.84)	0.660	1.43 (0.39–5.26)	0.592		
Tumor location						
Anterolateral	Ref.		Ref.			
Posterosuperior	1.06 (0.53–2.16)	0.864	0.43 (0.10–1.95)	0.275		
Tumor number						
<3	Ref.		Ref.		Ref.	
≥3	3.05 (1.31–7.08)	0.009	3.41 (1.11–10.44)	0.032	8.34 (1.70–40.92)	0.009
Tumor size (cm)						
<3.0	Ref.		Ref.			
≥3.0	0.85 (0.44–1.64)	0.620	0.40 (0.12–1.32)	0.131		
Tumor grade						
Good/moderate	Ref.		Ref.		Ref.	
Poor	2.00 (0.92–4.34)	0.079	8.57 (1.51–48.78)	0.015	17.98 (2.15–150.59)	0.008
Vascular invasion						
No	Ref.		Ref.		Ref.	
Yes	1.38 (0.53–3.60)	0.508	3.47 (1.04–11.55)	0.043	8.19 (1.54–43.45)	0.014
Resection status						
R0	Ref.		Ref.			
R1	2.80 (0.84–9.36)	0.095	5.11 (0.53–49.14)	0.158		

HR, hazard ratio; CI, confidence interval.

## Data Availability

The data presented in this study are available from the corresponding author upon request.
